# Association of IS*Mav6* with the Pattern of Antibiotic Resistance in Korean *Mycobacterium avium* Clinical Isolates but No Relevance between Their Genotypes and Clinical Features

**DOI:** 10.1371/journal.pone.0148917

**Published:** 2016-02-09

**Authors:** Su-Young Kim, Byeong-Ho Jeong, Hye Yun Park, Kyeongman Jeon, Seung Jung Han, Sung Jae Shin, Won-Jung Koh

**Affiliations:** 1 Division of Pulmonary and Critical Care Medicine, Department of Medicine, Samsung Medical Center, Sungkyunkwan University School of Medicine, Seoul, Korea; 2 Department of Microbiology, Institute for Immunology and Immunological Diseases, Brain Korea 21 PLUS Project for Medical Science, Yonsei University College of Medicine, Seoul, South Korea; Cornell University, UNITED STATES

## Abstract

The aim of this study was to genetically characterize clinical isolates from patients diagnosed with *Mycobacterium avium* lung disease and to investigate the clinical significance. Multi-locus sequencing analysis (MLSA) and pattern of insertion sequence analysis of *M*. *avium* isolates from 92 Korean patients revealed that all isolates were *M*. *avium* subspecies *hominissuis*. In *hsp65* sequevar analysis, codes 2, 15, and 16 were most frequently found (88/92) with similar proportions among cases additionally two isolates belonging to code N2 and an unreported code were identified, respectively. In insertion element analysis, all isolates were IS*1311* positive and IS*900* negative. Four of the *M*. *avium* subsp. *hominissuis* isolates did not harbor IS*1245* and 1 of the *M*. *avium* isolates intriguingly harbored DT1, which is thought to be a *M*. *intracellulare*-specific element. *M*. *avium* subsp. *hominissuis* harboring IS*Mav6* is prevalent in Korea. No significant association between clinical manifestation and treatment response has been found in patients with the *hsp65* code type and IS*Mav6*, indicating that no specific strain/genotype among *M*. *avium* subsp. *hominissuis* organisms was a major source of *M*. *avium* lung disease. Interestingly, the presence of IS*Mav6* was correlated with greater resistance to moxifloxacin. Conclusively, the genotype of Korean *M*. *avium* subsp. *hominissuis* isolates is not a disease determinant responsible for lung disease and specific virulent factors of *M*. *avium* subsp. *hominissuis* need to be investigated further.

## Introduction

A rise in the incidence of pulmonary disease caused by nontuberculous mycobacteria (NTM) has been reported worldwide [1, 2]. *Mycobacterium avium* complex (MAC) is the most frequent etiology of NTM lung disease [3]. MAC initially included two species, *M*. *avium* and *M*. *intracellulare*. *M*. *avium* is the most clinically significant species for humans and animals within the MAC and is divided into four subspecies: *M*. *avium* subsp. *avium*, *M*. *avium* subsp. *hominissuis*, *M*. *avium* subsp. *paratuberculosis*, and *M*. *avium* subsp. *silvaticum* [4, 5].

Although subspecies of *M*. *avium* in different geographic regions or populations may have different levels of virulence due to co-evolutionary processes, consequently leading to varying epidemiological dominance, most cases of *M*. *avium* human disease are due to *M*. *avium* subsp. *hominissuis*. Recently, lymphadenitis patients in France were found to be infected by only *M*. *avium* subsp. *hominissuis* among *M*. *avium* subspecies [6]. More recently, a subspecies identification analysis of *M*. *avium* clinical strains in the USA showed *M*. *avium* subsp. *hominissuis* to be the dominant *M*. *avium* subspecies (92.6%), followed by *M*. *avium* subsp. *avium* (7.4%) [7]. All German *M*. *avium* strains isolated from children and adults were identified as *M*. *avium* subsp. *hominissuis* [8].

Many studies have emphasized the importance of taxonomy in distinguishing species and subspecies of MAC because non-sequencing methods or 16S rRNA sequencing frequently fails to distinguish closely related species [9, 10]. Multi-locus sequencing analysis (MLSA) has been suggested as the new standard method for identifying *Mycobacterium* species that are not well discriminated by 16S rRNA gene sequences alone [11–14].

The presence and distribution of various insertion sequences (IS) among *M*. *avium* subspecies have provided an unprecedented opportunity to define the genomic differences between *M*. *avium* subspecies as well as to develop molecular typing methods with sufficient discriminatory power to differentiate *M*. *avium* subspecies and isolates [15].

At our institution, the *rpoB*-PCR restriction fragment length polymorphism (RFLP) analysis [PRA] method was used for species identification and diagnosis of MAC lung disease until 2009 [16–18]. To gain better insight into *M*. *avium* lung disease in Korea, we used sequencing-based methods for subspecies identification and genotyping and compared clinical characteristics and treatment outcomes according to genotype. Furthermore, we investigated patterns of antibiotic resistance according to mycobacterial genotype as well as the presence or absence of IS*Mav6*.

## Materials and Methods

### Study subjects

Clinical isolates from 92 patients with newly diagnosed *M*. *avium* lung disease from Jan. 2008 to Dec. 2009 at Samsung Medical Center (Seoul, Korea) were collected and stored. The data in the present study are part of an ongoing prospective observational cohort study investigating NTM lung disease (ClinicalTrials.gov Identifier: NCT00970801). The study protocol for isolates collection and genotyping analysis was approved by the institutional review board of the Samsung Medical Center (IRB approval 2008-09-016), and written informed consent was obtained from all participants. All patients met the diagnostic criteria for NTM lung disease [3]. All patients were immunocompetent and none of the patients tested positive for human immunodeficiency virus. All isolates were collected before initiating antibiotic treatment for NTM lung disease. Additionally, *M*. *avium* species initially identified by PRA based on the *rpoB* gene at the time of diagnosis as previously described were used for subsequent analysis.

### Drug susceptibility test

Drug susceptibility testing was performed at the Korean Institute of Tuberculosis. The minimum inhibitory concentrations (MICs) of clarithromycin (CLR) and moxifloxacin (MXF) were determined using the broth microdilution method as described by the Clinical and Laboratory Standards Institute (CLSI) [19]. Isolates with CLR MIC of ≥32 μg/ml and MXF MIC of ≥4 μg/ml were considered to be resistant, according to the guidelines of the CLSI [19].

### Molecular characterization of *M*. *avium* clinical isolates by MLSA

*M*. *avium* strains were propagated in Middlebrook 7H9 broth (Difco Laboratories, Detroit, MI, USA) supplemented with 10% (vol/vol) oleic acid-albumin-dextrose-catalase (OADC; BD Diagnostics, Sparks, MD, USA). Mycobacterial DNA was extracted using a DNeasy Blood and Tissue Kit according to the manufacturer’s instructions (Qiagen, Valencia, CA, USA). MLSA including *hsp65*, *rpoB*, and 16S rRNA fragments was carried out using PCR primer sets as described previously [20–22]. The PCR products of target genes were subjected to sequence analysis. The nucleotide sequences of these genes were compared with data reported by BLAST analysis (http://www.ncbi.nlm.nih.gov) against sequences from *M*. *avium* subspecies type and related strains. *M*. *avium* subsp. *avium* ATCC 25291, *M*. *avium* subsp. *hominissuis* 104, *M*. *avium* subsp. *paratuberculosis* K-10, and *M*. *avium* subsp. *silvaticum* ATCC 49884 were used as reference strains. For phylogenetic analysis, sequences were trimmed using the CLUSTAL-W multiple sequence alignment program [23]. Phylogenetic trees were obtained from DNA sequences utilizing the neighbor-joining method and Kimura’s two parameter distance correction model with 1,000 bootstrap replications supported by MEGA 6.0 software [24].

### hsp65 code analysis

*hsp65* code analysis was performed as previously described [25]. *hsp65* gene PCR products were subjected to sequence analysis. The nucleotide sequences of the *hsp65* gene were compared with data reported by BLAST analysis (http://www.ncbi.nlm.nih.gov) against the *M*. *avium* type and related strains. *hsp65* codes were classified according to previously reported papers [25–27].

### Insertion sequences element analysis

Multiplex PCR was performed to detect three target genes, IS*900*, IS*1311*, and DT1 using previously described methods [28]. A previously described primer set was used for the IS*1245* insertion element [29]. The presence of IS*Mav6* was determined by PCR followed by sequencing analysis using a previously described primer set [26]. PCR products of insertion elements were sequenced and the existence of a specific insertion element in each strain was confirmed. DNA isolated from *M*. *abscessus* ATCC 19977, *M*. *tuberculosis* H37Rv ATCC 27294, and *M*. *gastri* ATCC 15754 were used as negative controls for each primer set in each PCR run.

### Statistics

All data are presented as median and interquartile range for continuous variables and as number (percentage) for categorical variables. Data were compared using the Mann-Whitney *U* test for continuous variables and the chi-squared or Fisher’s exact test for categorical variables. The Mantel-Haenszel test for categorical variables was used to compare each *hsp65* code or the presence of IS*Mav*6 across resistant levels (susceptible, intermediate, resistant) of each drug [30]. Statistical analyses were performed using SAS 9.1 (SAS Institute Inc., Cary, NC, USA) and a *P*-value less than 0.05 was considered statistically significant.

## Results

### Subspecies identification of *M*. *avium* clinical isolates by MLSA

**Fig 1:**
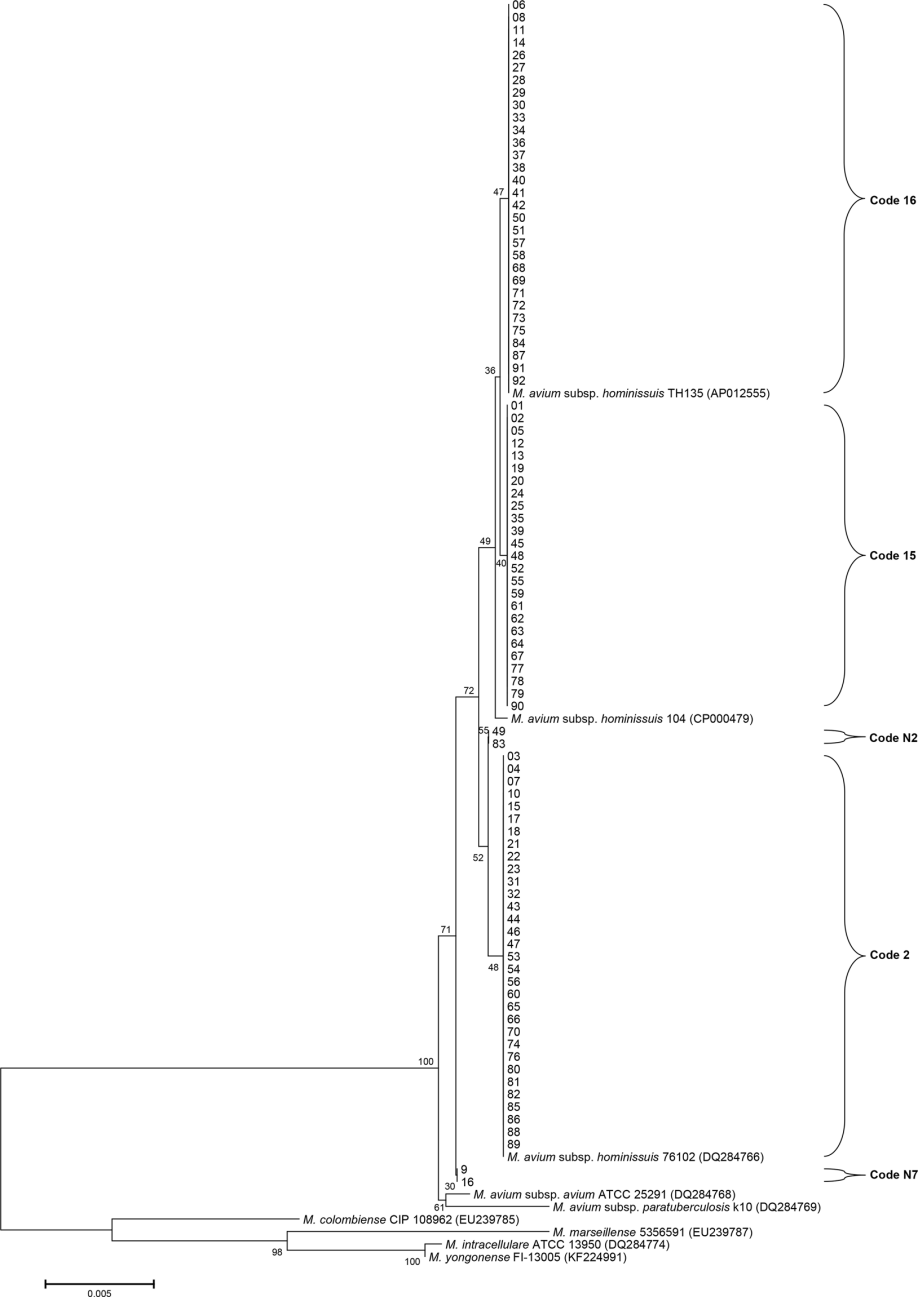
*hsp65* sequence-based phylogenetic tree using the neighbor-joining method with Kimura’s two-parameter distance correction model. Bootstrap analyses determined from 1,000 replicates are indicated at the nodes. Bar, 0.5% difference in nucleotide sequence. GenBank accession numbers are given in parentheses.

**Fig 2:**
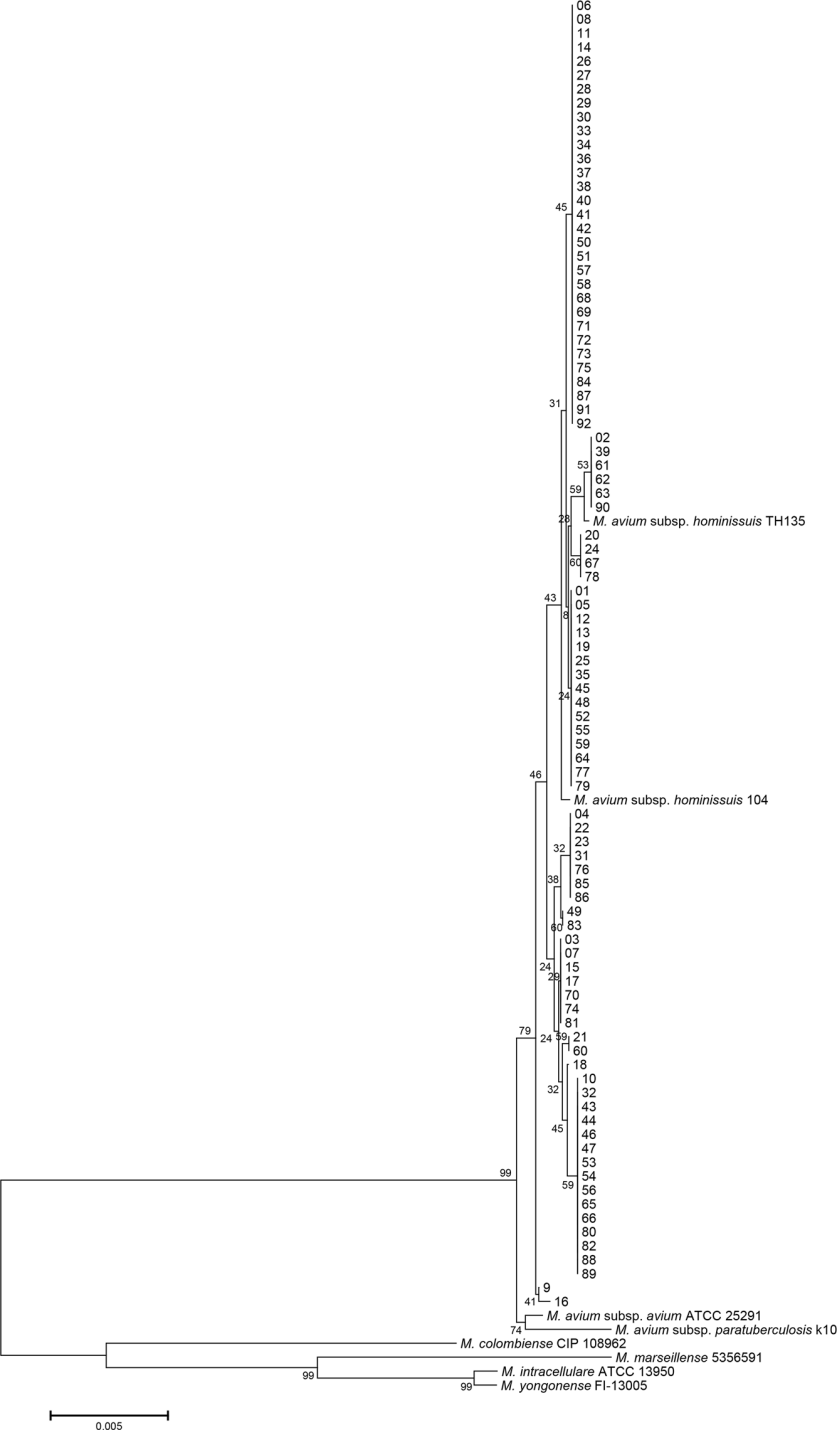
Phylogenetic tree based on concatenated *rpoB* and *hsp65* sequences using the neighbor-joining method with Kimura’s two-parameter distance correction model. Bootstrap analyses determined from 1,000 replicates are indicated at the nodes. Bar, 0.5% difference in nucleotide sequence. GenBank accession numbers are shown in Fig 1 and S1 Fig.

Isolates from 92 patients diagnosed with *M*. *avium* lung disease were re-identified. The 16S rRNA sequences of *M*. *avium* subsp. *hominissuis* are identical to those of the *M*. *avium* subsp. *avium*, *M*. *avium* subsp. *paratuberculosis*, and *M*. *avium* subsp. *silvaticum*. All isolates were identified as *M*. *avium* based on 16S rRNA sequencing. The *rpoB* sequences of 76 isolates were identical to those of the *M*. *avium* subsp. *hominissuis* type strain (GenBank accession no. CP000479) and clinical strain (GenBank accession no. AP012555) (S1 Fig). A total of 10 isolates showed a 1-bp mismatch (99.9% similarity) in a 711-bp fragment of the *rpoB* gene of *M*. *avium* subsp. *hominissuis* type strain (GenBank accession no. CP000479). The other 6 isolates were identical to those of the *M*. *avium* subsp. *avium* type strain (GenBank accession no. GQ153306). However, the nearly complete *hsp65* sequences of all isolates, except 4 isolates, were identical only to those of the *M*. *avium* subsp. *hominissuis* type strain (GenBank accession no. CP000479) and clinical strains (GenBank accession nos. AP012555 and DQ284766) (Fig 1). Each pair of the 4 isolates were 99.8% (1413/1416) and 99.7% (1412/1416) similar to the *hsp65* sequences of the *M*. *avium* subsp. *hominissuis* type strain (GenBank accession no. CP000479). The *hsp65* sequences of *M*. *avium* subsp. *hominissuis* were different (6-bp mismatches) from those of the *M*. *avium* subsp. *avium* type strain (GenBank accession no. DQ284768). Phylogenetic analysis based on the concatenated *hsp65* and *rpoB* sequences from all isolates and from those of closely related species within the MAC showed that all isolates belong to *M*. *avium* subsp. *hominissuis* (Fig 2). Therefore, all isolates were identified as *M*. *avium* subsp. *hominissuis* using MLSA.

### Distribution of *hsp65* codes in *M*. *avium* subsp. *hominissuis* strains

In total, the 92 isolates were classified into five different *hsp65* sequevars. There were no isolates classified to *hsp65* code 4, the *M*. *avium* subsp. *avium* sequevar. Four of these sequevars were well recognized as *M*. *avium* subsp. *hominissuis* type and clinical strains and 1 sequevar code was newly identified in this study. The new sequevar was coded N7 (following the code name given in the previous paper [N1-N3] [26] and accepted paper [N4-N6]) and two isolates were classified as code N7. The distribution of *hsp65* sequevars in the 92 isolates is shown in Table 1. The major codes were 2, 15, and 16.

**Table 1:** Identification of the novel *hsp65* sequevar code and distribution of *hsp65* codes and insertion elements in this study.

*hsp65* code^a^	Nucleotide at the indicated base pair position (*hsp65*)^b^									*n* (%)	*n* (%) of isolates				
	324	645	927	1128	1218	1269	1272	1350	1536		IS*900*	IS*1245*	IS*1311*	DT1	IS*Mav6*
Code 2	T	C	C	C	G	G	C	G	G	32 (35)	0	32	32	0	15 (47)
Code 15	C	•	T	•	A	•	•	•	A	25 (27)	0	22	25	0	21 (84)
Code 16	C	•	T	•	A	•	•	•	•	31 (34)	0	31	31	0	18 (58)
Code N2	•	•	T	•	•	•	•	•	•	2 (2)	0	1	2	0	1 (50)
Code N7^c^	C	•	•	•	•	•	G	•	•	2 (2)	0	2	2	1	1 (50)
Total										92 (100)	0	88	92	1	56 (61)

^a^ Classification according to previously reported papers [25–27].

^b^ • indicates the same base pair as in code 2.

^c^ New code type found in this study designated code N7.

### Detection of insertion sequence elements

All isolates were negative for IS*900* (considered diagnostic for *M*. *avium* subsp. *paratuberculosis*) [31] and positive for IS*1311* (considered diagnostic for all members of *M*. *avium* subspecies) [32]. One isolate belonging to the new sequevar N7 possessed DT1 (considered diagnostic for *M*. *intracellulare* and *M*. *avium* subsp. *avium*); the DT1 sequence of this isolate was similar (92% and 89% similarity) to those of the *M*. *intracellulare* clinical strain and *M*. *avium* serotype 2 (*M*. *intracellulare*) DT1 (GenBank accession nos. CP003491 and L04543), suggesting that horizontal transfer of DT1 might occur from *M*. *intracellulare* to *M*. *avium*. Among 92 isolates, 88 (96%) were positive for IS*1245* (considered to be limited to *M*. *avium* subsp. *avium*, *M*. *avium* subsp. *hominissuis*, and *M*. *avium* subsp. *silvaticum)* [5, 33, 34] and the other 4 isolates did not harbor IS*1245*. Interestingly, 56 (61%) isolates possessed IS*Mav6*. The code 15 sequevar showed a significantly higher prevalence of IS*Mav6* (84%) than did the other codes (Table 1).

### Relatedness of clinical characteristics, treatment response, and drug susceptibility to *hsp65* codes and presence/absence of IS*Mav6*

We analyzed clinical characteristics and treatment response among 3 major codes (code 2, 15, and 16). There were no significant differences in clinical features among the 3 groups (S1 and S2 Tables). We also analyzed clinical characteristics and treatment response according to the presence of IS*Mav6*. There were no significant differences in clinical features between those with and without IS*Mav6* (S3 and S4 Tables). The association of genotype and the presence of IS*Mav6* with drug susceptibility patterns in the *M*. *avium* subsp. *hominissuis* isolates was evaluated for CLR and MXF. Drug susceptibility test were performed in 72 and 71 patients for CLR and MXF, respectively. None of the *hsp65* codes showed trends in drug susceptibility levels (data not shown); however, the presence of IS*Mav6* was correlated with greater resistance to MXF (Table 2).

**Table 2:** Association between presence of IS*Mav6* and antibiotic resistance.

Drug susceptibility test	Detection of IS*Mav6*		*P*-value for trend test
	IS*Mav6* (+)	IS*Mav6* (-)	IS*Mav6* (+) *vs*. IS*Mav6* (-)
CLR			0.375
S	42	27	
I	1	0	
R	2	0	
MXF			0.003
S	17	17	
I	9	9	
R	18	1	

Data are presented as numbers.

Because drug susceptibility testing was not performed in all patients, the sum of the individual categories is less than 92.

Definition of abbreviations: CLR = clarithromycin; MXF = moxifloxacin; S = susceptible; I = intermediate; R = resistant.

## Discussion

In this study, clinical isolates from 92 patients previously diagnosed with *M*. *avium* lung disease over a two-year period were further analyzed. Species identification was initially performed by a non-sequencing method and then species were re-identified using a sequencing method. Among the 92 isolates identified as *M*. *avium* by PRA at the time of diagnosis, all isolates were precisely identified as *M*. *avium* subsp. *hominissuis*.

In *hsp65* sequevar analysis, the major codes were 2, 15, and 16 (35%, 27%, and 34%, respectively). Among Japanese clinical isolates, the main codes were 2 (63/146, 43%) and 15 (50/146, 34%), followed by 16 (9/146, 6%) [26]. The prevalence of code 16 in Korea was higher than in Japan.

IS*Mav6* is a novel IS recently reported in the genetic characterization of Japanese human clinical isolates [27]. In the present study, the prevalence of IS*Mav6* in Korean patients with *M*. *avium* lung disease was 61%. Interestingly, more clinical isolates with *hsp65* code 15 harbored IS*Mav6* (84%, 21/25) than isolates with *hsp65* code 2 and code 15 (47% and 58%, respectively). Also, both *hsp65* code 15 and IS*Mav6* have rarely been reported in the literature except in Japan. In Germany, one *M*. *avium* subsp. *hominissuis* strain with *hsp65* code 15 harboring IS*Mav6* was reported [8]. The high proportion of IS*Mav6* in *M*. *avium* subsp. *hominissuis* strains from Korea and Japan is thought to be a specific genetic feature. Thus, both *hsp65* code 15 and IS*Mav6* may be related to the epidemiological diversity of *M*. *avium* clinical strains.

In general, DT1 is present in *M*. *intracellulare* and not present in *M*. *avium* subsp. *hominissuis*. One *M*. *avium* subsp. *hominissuis* isolates possessed DT1 in this study, which is a novel observation. Since a number of different IS elements have been described in various NTM species, species-specific IS elements have been revisited for MAC identification [28, 29, 35]. IS elements are mobile by nature, so there is a risk that similar elements will be found in unrelated bacteria because of mobility to or from MAC organisms. For example, natural occurrence of horizontal transfer of *M*. *avium*-specific IS*1245* to *M*. *kansasii* has been reported [36]. Thus, the use of insertion sequences for species-specific markers should be more carefully conducted because it may influence molecular diagnosis and, consequently, treatment outcomes.

Kikuchi et al. reported that a variable number of tandem repeats (VNTR)-genotyping of 37 *M*. *avium* clinical isolates was associated with the progression of *M*. *avium* lung disease in Japan [37]. However, our study, which included more than 100 clinical isolates, did not identify any association between the *M*. *avium* VNTR genotype and disease progression of *M*. *avium* lung disease [38]. In the present study, disease progression was defined as when patients with *M*. *avium* lung disease require antibiotic treatment due to worsening symptoms, deteriorating chest radiograph features and microbiological findings within 2 years of diagnosis [39].

There was no difference in clinical characteristics and treatment response according to *hsp65* sequevar codes and IS*Mav6*, in agreement with previous VNTR-based observations that there was no association between the genotype and clinical characteristics of Korean patients [38]. Interestingly, the presence of IS*Mav6* was associated with drug resistance to MXF in this study. Tatano et al. reported an association between the VNTR genotype and susceptibility to quinolones and EMB [40]. Dvorska et al. found no relationship between IS*1311* and IS*1245*-based RFLP genotypes and drug susceptibility in MAC isolates [41]. These findings suggest that some genetic factors may influence the acquisition of drug resistance and IS*Mav6* may be a genetic factor associated with drug resistance. As far as we know, this is the first study to suggest an association between genotypes according to *hsp65* codes and IS*Mav6* and clinical features with drug susceptibility. Our results indicate that specific genotypes among *M*. *avium* subsp. *hominissuis* organisms are not predominantly responsible for *M*. *avium* lung disease in Korea and further analysis of IS*Mav6* (*i*.*e*. the location of IS*Mav6* in the genome of *M*. *avium* isolates) will help identify relationships between genetic features and drug susceptibility.

The present study has some limitations. First, this study was conducted at a single center and was performed on a referral basis with final analysis of only a small number of Korean patients; therefore, caution should be used when attempting to generalize our findings. Second, this study was preliminary because we did not investigate the specific genes associated with drug resistance. Thus, further precise drug resistance typing of *rpoB* and *gyrA/B* with a large number of isolates will provide a better understanding of the association between *M*. *avium* subsp. *hominissuis* genotypes and drug resistance.

Nevertheless, to the best of our knowledge, this is the first report to investigate the link between IS*Mav6* and drug resistance to MXF in *M*. *avium* subsp. *hominissuis* strains from Korean patients. Future studies of informative and valuable genetic factors related to *M*. *avium* lung disease should be conducted in both the pathogen and host.

## Supporting Information

S1 Fig *rpoB* sequence-based phylogenetic tree using the neighbor-joining method with Kimura’s two-parameter distance correction model. Bootstrap analyses determined from 1,000 replicates are indicated at the nodes. Bar, 0.5% difference in nucleotide sequence. GenBank accession numbers are given in parentheses.
(TIF)

S1 Table Clinical characteristics according to *hsp65* sequevar codes. (DOC)

S2 Table Treatment response according to *hsp65* sequevar codes. (DOC)

S3 Table Clinical characteristics according to the presence or abscece of IS*Mav6*. (DOC)

S4 Table Treatment response according to the presence or absence of IS*Mav6*. (DOC)

## Abbreviations

MAC: *Mycobacterium avium* complex
MLSA: multi-locus sequencing analysis
IS: insertion sequences
RFLP: restriction fragment length polymorphism
PRA: PCR restriction fragment length polymorphism analysis
NTM: non-tuberculous mycobacteria
CLM: clarithromycin
RIF: rifampin
EMB: ethambutol
MXF: moxifloxacin
MIC: minimum inhibitory concentration
CLSI: Clinical and Laboratory Standards Institute
VNTR: variable number of tandem repeats
