# Supplementary figures and images for: Association of ISMav6 with the Pattern of Antibiotic Resistance in Korean Mycobacterium avium Clinical Isolates but No Relevance between Their Genotypes and Clinical Features
Source: PLoS One. 2016 Feb 9;11(2):e0148917. doi: 10.1371/journal.pone.0148917 (PMC4747469; doi:10.1371/journal.pone.0148917)

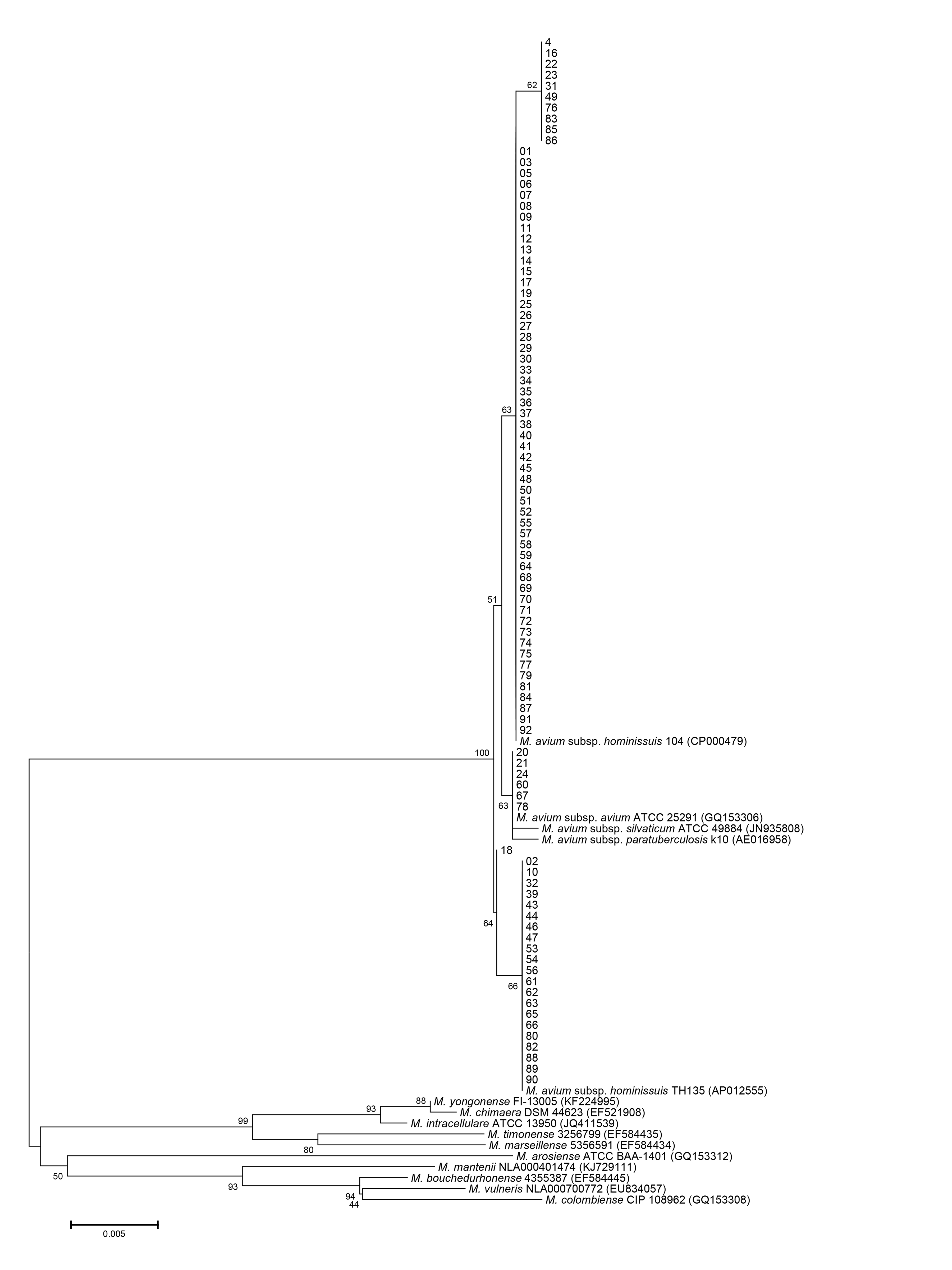

Supplement: S1 Fig — Bootstrap analyses determined from 1,000 replicates are indicated at the nodes. Bar, 0.5% difference in nucleotide sequence. GenBank accession numbers are given in parentheses. (TIF) [file pone.0148917.s001.tif]
