# Supplementary material for: Association of ISMav6 with the Pattern of Antibiotic Resistance in Korean Mycobacterium avium Clinical Isolates but No Relevance between Their Genotypes and Clinical Features
Source: PLoS One. 2016 Feb 9;11(2):e0148917. doi: 10.1371/journal.pone.0148917 (PMC4747469; doi:10.1371/journal.pone.0148917)
Supplement: S1 Table — (DOC) [file pone.0148917.s002.doc]

**Table S1** Clinical characteristics according to *hsp65* sequevar codes

| Characteristic | *hsp65* code 2  (*n* = 32) | *hsp65* code 15  (*n* = 25) | *hsp65* code 16  (*n* = 31) | *P*-value |
| --- | --- | --- | --- | --- |
| Male, sex | 15 (47) | 14 (56) | 18 (58) | 0.642 |
| Age, year | 59 (52-66) | 57 (46-63) | 62 (48-69) | 0.320 |
| BMI, kg/m^2^ | 20.1 (19.0-21.3) | 20.8 (19.6-21.9) | 20.6 (18.8-22.5) | 0.505 |
| Nonsmoker | 19 (59) | 17 (68) | 20 (65) | 0.326 |
| Associated diseases |  |  |  |  |
| Previous TB | 10 (31) | 9 (36) | 9 (29) | 0.853 |
| Bronchiectasis | 24 (75) | 16 (64) | 23 (74) | 0.608 |
| COPD | 2 (6) | 4 (16) | 1 (3) | 0.194 |
| IPF | 0 | 0 | 1 (3) | 0.395 |
| Malignancy | 4 (13) | 5 (20) | 6 (19) | 0.691 |
| Chronic heart disease | 6 (19) | 5 (20) | 5 (16) | 0.928 |
| Diabetes mellitus | 6 (19) | 2 (8) | 3 (10) | 0.400 |
| Chronic liver disease | 0 (0) | 1 (4) | 1 (3) | 0.547 |
| Symptoms |  |  |  |  |
| Cough | 23 (72) | 18 (72) | 23 (74) | 0.974 |
| Sputum | 21 (66) | 18 (72) | 21 (68) | 0.875 |
| Hemoptysis | 8 (25) | 6 (24) | 5 (16) | 0.653 |
| Weight loss | 0 | 0 | 1 (3) | 0.395 |
| Fever | 0 | 1 (4) | 0 | 0.280 |
| Laboratory findings |  |  |  |  |
| Positive AFB smear | 11 (34) | 12 (48) | 16 (52) | 0.352 |
| ESR, mm/h | 20.0 (12.0-28.0) | 24.5 (14.0-75.8) | 16.0 (8.0-31.0) | 0.234 |
| CRP, mg/L | 0.8 (0.3-1.5) | 1.1 (0.5-19.9) | 1.3 (0.4-7.9) | 0.311 |
| Type of disease |  |  |  | 0.886 |
| Fibrocavitary | 7 (22) | 5 (20) | 5 (16) |  |
| Nodular bronchiectatic | 22 (69) | 16 (64) | 23 (74) |  |
| Unclassifiable | 3 (9) | 4 (16) | 3 (10) |  |

Data are presented as number (%) or median (interquartile range).

Definition of abbreviations: BMI=body mass index; TB=tuberculosis; COPD=chronic obstructive pulmonary disease; IPF=idiopathic pulmonary fibrosis; AFB=acid-fast bacillus; ESR=erythrocyte sedimentation rate; CRP=C-reactive protein.
