# Supplementary material for: Association of ISMav6 with the Pattern of Antibiotic Resistance in Korean Mycobacterium avium Clinical Isolates but No Relevance between Their Genotypes and Clinical Features
Source: PLoS One. 2016 Feb 9;11(2):e0148917. doi: 10.1371/journal.pone.0148917 (PMC4747469; doi:10.1371/journal.pone.0148917)
Supplement: S2 Table — (DOC) [file pone.0148917.s003.doc]

**Table S2** Treatment response according to *hsp65* sequevar codes

|  | *hsp65* code 2  (*n* = 18) | *hsp65* code 15  (*n* = 15) | *hsp65* code 16  (*n* = 22) | *P*-value |
| --- | --- | --- | --- | --- |
| Initiation of antibiotic therapy | 18 (56) | 15 (60) | 22 (71) | 0.461 |
| Treatment responses after 12 mon of treatment  Symptomatic improvement  Radiologic improvement  Sputum conversion | 11 (79)  10 (56)  12 (67) | 7 (50)  10 (67)  10 (67) | 16 (80)  12 (55)  11 (50) | 0.125  0.283  0.403 |
| Final outcome with antibiotic therapy  Therapy success  Therapy failure  Death  Discontinuation of antibiotics^*^ | 13 (72)  1 (6)  1 (6)  3 (17) | 10 (67)  3 (20)  0 (0)  2 (13) | 13 (59)  7 (32)  0 (0)  2 (9) | 0.403 |

Data are presented as number (%).

*Due to adverse effects (*n* = 5) or other serious comorbidities (*n* = 2)
