# Supplementary material for: Association of ISMav6 with the Pattern of Antibiotic Resistance in Korean Mycobacterium avium Clinical Isolates but No Relevance between Their Genotypes and Clinical Features
Source: PLoS One. 2016 Feb 9;11(2):e0148917. doi: 10.1371/journal.pone.0148917 (PMC4747469; doi:10.1371/journal.pone.0148917)
Supplement: S3 Table — (DOC) [file pone.0148917.s004.doc]

***Table S3*** *Clinical characteristics according to the presence or absence of ISMav6*

| Characteristic | IS*Mav6* (+)  (*n* = 56) | IS*Mav6* (-)  (*n* = 36) | *P*-value |
| --- | --- | --- | --- |
| Male, sex | 31 (55) | 17 (47) | 0.446 |
| Age, year | 59 (50 - 68) | 59 (50 - 66) | 0.689 |
| BMI, Kg/m2 | 20.6 (19.6 - 22.4) | 20.1 (18.7 - 21.3) | 0.142 |
| Nonsmoker | 32 (57) | 27 (75) | 0.205 |
| Associated diseases |  |  |  |
| Previous TB | 15 (27) | 13 (36) | 0.343 |
| Bronchiectasis | 41 (73) | 26 (72) | 0.917 |
| COPD | 4 (7) | 3 (8) | 0.834 |
| IPF | 0 (0) | 1 (3) | 0.391 |
| Malignancy | 7 (13) | 9 (25) | 0.123 |
| Chronic heart disease | 11 (20) | 6 (17) | 0.720 |
| Diabetes mellitus | 5 (9) | 7 (19) | 0.205 |
| Chronic liver disease | 2 (4) | 1 (3) | 1.000 |
| Symptoms |  |  |  |
| Cough | 37 (66) | 28 (78) | 0.229 |
| Sputum | 37 (66) | 24 (67) | 0.953 |
| Hemoptysis | 13 (23) | 6 (17) | 0.449 |
| Weight loss | 1 (2) | 0 (0) | 1.000 |
| Fever | 1 (2) | 0 (0) | 1.000 |
| Laboratory findings |  |  |  |
| Positive AFB smear | 25 (45) | 15 (42) | 0.779 |
| ESR, mm/h | 22 (11-32) | 20 (10-45) | 0.872 |
| CRP, mg/L | 1.00 (0.03 – 0.34) | 0.80 (0.40-5.23) | 0.832 |
| Type of disease |  |  | 0.746 |
| Fibrocavitary | 8 (22) | 9 (16) |  |
| Nodular bronchiectatic | 24 (67) | 41 (73) |  |
| Unclassifiable | 4 (11) | 6 (11) |  |

Data are presented as number (%) or median (interquartile range).

Definition of abbreviations: BMI=body mass index; TB=tuberculosis; COPD=chronic obstructive pulmonary disease; IPF=idiopathic pulmonary fibrosis; AFB=acid-fast bacillus; ESR=erythrocyte sedimentation rate; CRP=C-reactive protein.
