# Supplementary material for: Association of ISMav6 with the Pattern of Antibiotic Resistance in Korean Mycobacterium avium Clinical Isolates but No Relevance between Their Genotypes and Clinical Features
Source: PLoS One. 2016 Feb 9;11(2):e0148917. doi: 10.1371/journal.pone.0148917 (PMC4747469; doi:10.1371/journal.pone.0148917)
Supplement: S4 Table — (DOC) [file pone.0148917.s005.doc]

**Table S4** Treatment response according to the presence or absence of IS*Mav6*

|  | IS*Mav6* (+)  (*n* = 35) | IS*Mav6* (-)  (*n* = 21) | *P*-value |
| --- | --- | --- | --- |
| Initiation of antibiotic therapy | 35 (63) | 21 (58) | 0.689 |
| Treatment responses after 12 mon of treatment  Symptomatic improvement  Radiologic improvement  Sputum conversion | 20 (57)  21 (60)  21 (60) | 15 (71)  12 (57)  13 (62) | 0.354  0.707  0.280 |
| Final outcome with antibiotic therapy  Therapy success  Therapy failure  Death  Discontinuation of antibiotics^*^ | 22 (63)  7 (20)  0 (0)  6 (18) | 15 (71)  4 (19)  1 (5)  1 (5) | 0.330 |

Data are presented as number (%).

*Due to adverse effects (*n* = 5) or other serious comorbidities (*n* = 2)
